# Effect of three-segment scanning on the accuracy of intraoral scans in full-arch implant-supported rehabilitation: an in vitro study

**DOI:** 10.1186/s40729-026-00692-4

**Published:** 2026-05-08

**Authors:** Yuki Sato, Yusuke Uto, Tetsurou Odatsu, Masami Arai, Takashi Sawase

**Affiliations:** 1 Private Practice, 5-18 Nokendai-dori, Kanazawa-ku, Yokohama, 236-0053 Japan; 2 Department of Applied Prosthodontics, Institute of Biomedical Sciences, Nagasaki University, 1-7-1, Sakamoto, Nagasaki, 852-8588 Japan; 3 Private Practice, 1-1-15 Kita-Shinagawa, Shinagawa-ku, Tokyo, 140-0001 Japan

**Keywords:** Digital scan, Intraoral scanner, Segmented impression, Accuracy, Edentulous, Implant

## Abstract

**Purpose:**

To evaluate the effect of three-segment scanning on the accuracy of digital impressions of full-arch implants obtained using intraoral scanners.

**Methods:**

An edentulous master model with six implants was digitized using a laboratory scanner to create a reference scan. Three intraoral scanners (Primescan, Aoralscan 3 and Trios 5) and two scan strategies were evaluated (*n* = 10): full-scan (simultaneous full-arch scan) and segmented-scan (the arch categorized into three segments, scanned independently, and subsequently composed into a full-arch dataset) groups. The reference data were scanned using a laboratory scanner. Surface deviation was evaluated with a tolerance of ± 50 μm. The percentage of the total scan body surface area within this tolerance range was calculated as surface trueness. The overall distance deviation between scan bodies was measured to determine linear trueness and precision.

**Results:**

For Aoralscan 3 and Trios 5, the segmented-scan group showed higher surface trueness than the full-arch scan group, whereas Primescan showed no statistically significant difference between the scan strategies. The overall distance deviation showed no significant differences in trueness between the full-arch and segmented scans for Primescan, Aoralscan 3, or Trios 5 (all *p* > 0.05). However, precision was significantly higher under the segmented-scan condition than with full-arch scanning for all evaluated intraoral scanners (all *p* < 0.01).

**Conclusions:**

For full-arch implant impressions using intraoral scanners, segmented scanning with subsequent composition into a full-arch digital model improved intraoral scanning accuracy compared with simultaneous full-arch scanning.

## Background

Full-arch implant-supported prostheses are a viable treatment option for edentulous patients [1, 2]. The fabrication of implant-supported prostheses involves several steps. Accurate impression making and transfer of the implant position to the working model are essential for fabricating a passively fitting prosthesis. However, low impression precision and other errors in clinical and technical workflows can result in misfit, leading to subsequent biological and mechanical complications [3]. The conventional workflow, which involves physical impressions and definitive stone casts, involves various factors associated with prosthesis inaccuracies. Ongoing chemical reactions, deformation of the impression material, time interval between impression taking and pouring, wettability of dental stone, and volume change of dental stone may cause distortion of impression materials and compromise impression accuracy [4]. Therefore, an open-tray technique with splinted copings is recommended for multiple edentulous implant impressions [5].

Digital technology offers advantages such as increased patient comfort and accurate registration of implant positions using scan bodies during intraoral scanning [6]. For single- or three-unit prostheses, digital scans using an intraoral scanner (IOS) and scan bodies have shown accuracy comparable to that of conventional impressions [7]. However, the accuracy of full-arch digital scans remains controversial [8].

Several studies have documented the factors affecting digital scan accuracy; the quality of a digital scan depends on the accuracy and precision of the IOS [9], operator experience [10], and the scanning strategy adopted by the operator [11]. Stitching error is one of the most important variables for assessing the accuracy of digital scans. IOSs merge thousands of images acquired during the scanning process, which can result in stitching errors, particularly in edentulous patients, as identifiable landmarks and stable reference points on the mucosal surfaces between the inter-implants remain unknown [12]. Inevitable errors occur during each overly progressive process, and cumulative errors may lead to unacceptable deviations as the scan area increases [13].

Various methods have been used to reduce stitching errors during the scanning of edentulous arches with implants, including creating reference points on the mucosal surface [14], modifying the design of scan bodies [15], and using splinted scan bodies [16]. Although these methods have improved scan accuracy to a certain extent, limitations include complex operation procedures, time-consuming steps, and additional scanning [17].

In this study, to reduce stitching errors during digital scans using IOS and scan bodies, segmented scanning was performed and composed using computer-aided design (CAD) software to create a model of edentulous scanning. This in vitro study aimed to evaluate the effects of three-segment scanning on digital scan accuracy. The null hypothesis was that no difference in accuracy would be observed between segmented and full-arch digital scans.

## Methods

### Preparation of the implant model

**Fig. 1:**
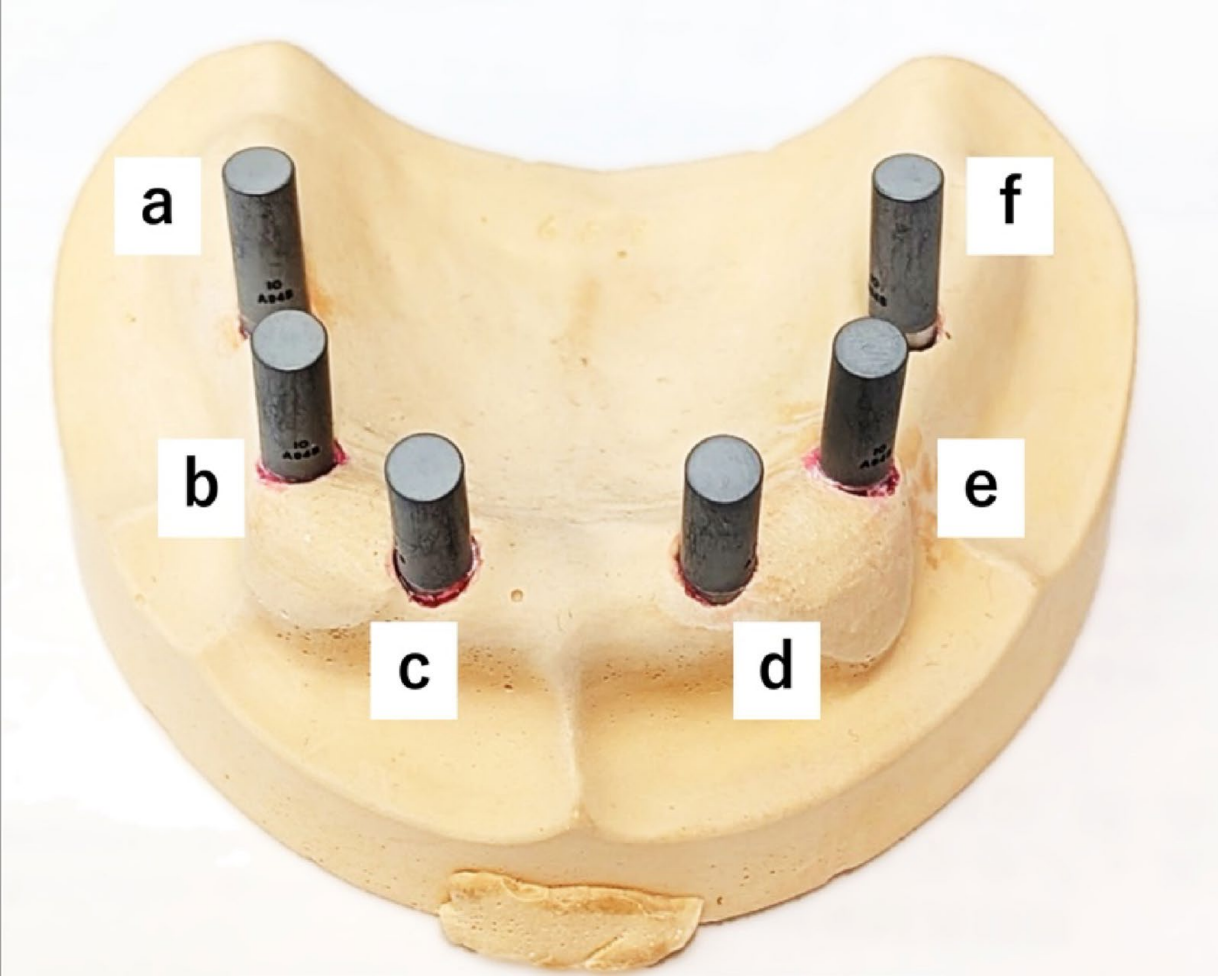
Implant model and tightened abutment-level scan bodies. A partially edentulous maxillary stone model was customized

A partially edentulous maxillary stone model (E3-599; Nissin Dental Products, Inc., Kyoto, Japan) was used as the master model. Six implant abutment analogs (Multibase EV Replica; Dentsply Sirona, Charlotte, NC, USA) were situated on both sides of the lateral incisors, first premolars, and first molars, with reference to a dentulous maxillary stone model (E3-500 A-U; Nissin Dental Products Inc.). Analogs in the first molar positions were aligned vertically relative to the occlusal plane. Analogs of the first premolars and lateral incisors were implanted at 5° and 10°, respectively, relative to the first molar analogs. The remaining teeth in the partially edentulous model were ground to create an edentulous model (Fig. 1).

### Scanning procedures

To obtain a digital reference model, abutment-level scan bodies (FLO, Atlantis IOFLO-S, Dentsply Sirona) were tightened to each analog following the recommendations of the manufacturer. The model was then digitized using a laboratory scanner (X5, inEosX5, Dentsply Sirona). The scanned dataset was exported in Standard Tessellation Language format and used as the reference for measurement. The multiple scans were taken with a laboratory scanner, and the datasets were confirmed to be more than 99.9% consistent within a surface tolerance of ± 50 μm. One of these was randomly selected as the reference data.

Three IOSs were used for the test groups: PS (Primescan, Connect SW version 5.2; Dentsply Sirona, Bensheim, Germany), AOS (Aoralscan 3 Wireless, IntraoralScan software version 3.4.1.7; Shining 3D Tech Co., Ltd., Hangzhou, China), and TRIOS (TRIOS 5, 3Shape Unite software version 25.1.0; 3Shape A/S, Copenhagen, Denmark). Full-arch and three-segment scans were performed by an experienced operator under the same humidity and temperature conditions.

For full-arch scanning, the scan started on the right side and proceeded to the left, capturing the palatal side. Subsequently, the scan was performed from left to right, passing exclusively through the buccal part, and additional scanning was performed to fill the branched area.

For three-segment scanning, the scanning area was divided into three segments: the right side (three scan bodies labeled d, e, and f in Fig. 1), left side (three scan bodies labeled a, b, and c in Fig. 1), and center (four scan bodies labeled b, c, d, and e in Fig. 1). Each segment was scanned using the same procedure as that used for the full-arch scan.

### Data processing and analysis

**Fig. 2:**
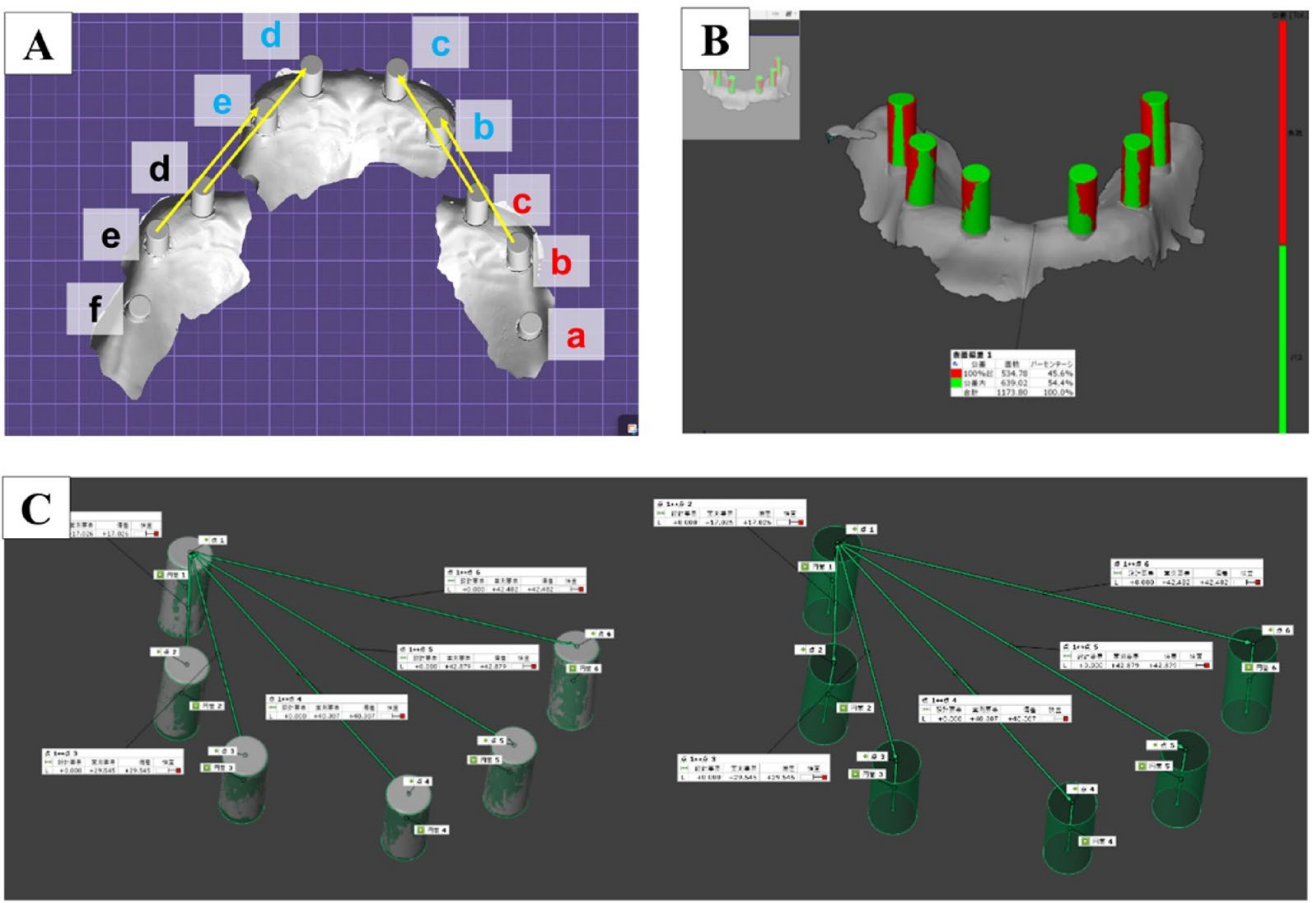
Evaluation workflow for surface and distance accuracies: (**A**) Merging procedure for segment scans. Individually acquired segment datasets were aligned and combined to reconstruct a complete arch model. (**B**) Surface deviation analysis between the reference and test datasets after best-fit alignment. The color maps represent deviations within a tolerance of ± 50 μm. (**C**) Distance-based accuracy evaluation: Cylindrical features were fitted to each scan body, and the differences in inter-scan-body distances (Δ*d*) and overall distance deviation (Δ*d*_overall_) were calculated to assess the trueness and precision

To compose the segmented scanning data, exocad DentalCAD software (exocad DentalDB 3.1 Rijeka 8200, Darmstadt, Germany) with a best-fit algorithm was used. The datasets from both sides, each containing three scan bodies, were matched with the central dataset, which contained four scan bodies. The central segment was set as the fixed mesh, and the left and right segments as floating meshes for “mesh registration.” After specifying two corresponding points on the scan body for initial alignment, fine adjustments were made using best-fit matching. Subsequently, redundant overlapping regions were removed to generate a single, unified dataset (Fig. 2A).

To compare the accuracy of each group with the reference data, ZEISS Inspect diagnostic software (version 2025; ZEISS Industrial Quality Solutions, Braunschweig, Germany) was employed using a best-fit algorithm [18]. The CAD file served as the reference, whereas the mesh file served as the test dataset. The realignment function was used for superimposition. In the reference dataset, only the scan body regions were retained for analysis. After alignment, surface deviation was evaluated and visualized using a color map at a tolerance of ± 50 μm. The percentage of the total scan body surface area within this tolerance range was calculated as surface trueness(Fig. 2B) [19].

Furthermore, to measure the distances between scan bodies a–b, a–c, a–d, a–e, and a–f, a geometrically fitted cylinder was applied to each scan body [20]. The intersection point between the central axis of the fitted cylinder and top surface of the scan body was defined as the measurement point (Fig. 2C). For each pair, the deviation was calculated as the difference between the measured and corresponding distances in the reference dataset (Δ*d*_ab_, Δ*d*_ac_, Δ*d*_ad_, Δ*d*_ae_, and Δ*d*_af_). The overall deviation (Δ*d*_*overall*_) was obtained as the sum of these individual distance deviations and represents as the linear trueness.

The deviation of each inter-scan body distance was calculated as:

$$ \Delta d_{{{\mathrm{ij}}}} = \left| {d_{{{\mathrm{ij}}}}^{{test}} - d_{{{\mathrm{ij}}}}^{{reference}} } \right| $$

where i and j denote the corresponding scan body positions.

The overall distance deviation (Δ*d*_*overall*_) was calculated as the sum of the absolute deviations of all inter-scan-body distances:

$$ \Delta d_{{overall}} = \Delta d_{{{\mathrm{ab}}}} + \Delta d_{{{\mathrm{ac}}}} + \Delta d_{{{\mathrm{ad}}}} + \Delta d_{{{\mathrm{ae}}}} + \Delta d_{{{\mathrm{af}}}} $$

Precision was defined as intra-group variability.

Absolute differences between all possible pairs were calculated for each group (*n* = 10).

$$ \left| {\Delta d_{{{\mathrm{overall,i}}}} - \Delta d_{{{\mathrm{overall,j}}}} } \right| $$

Total number of combinations: C^2^_10_ = 45.

The mean of these pairwise absolute differences represents the precision.

### Statistical analysis

Data are presented as the mean ± standard deviation. All statistical analyses were performed using JMP software (version 18; SAS Institute Inc., Cary, NC, USA). The normality of the data was assessed using the Shapiro–Wilk test.

Surface trueness and linear trueness (Δd _overall_) between the full-arch and segment-scan conditions were compared using Student’s t-test. Precision, calculated as the mean absolute intragroup pairwise differences in Δd _overall_, was compared between scan conditions using the Mann–Whitney U test. Statistical significance was set at *p* = 0.05.

## Results

### Surface trueness

**Fig. 3:**
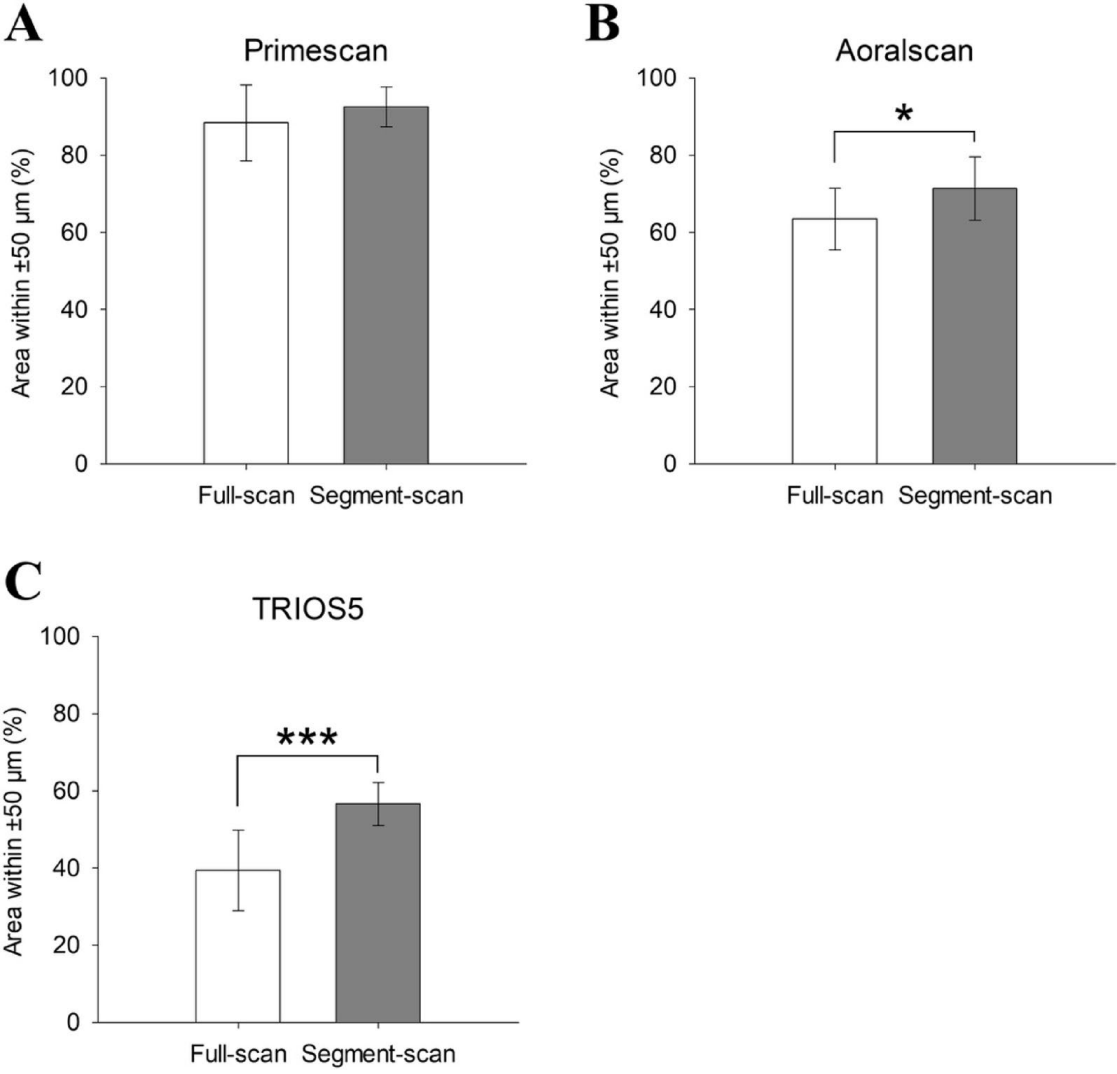
Surface trueness under full-arch and segment-scan conditions. The percentage of the intaglio surface area within a tolerance of ± 50 μm after best-fit alignment to the reference model is shown. Bars represent mean ± standard deviation (*n* = 10 per group). (**A**) Primescan (PS). (**B**) Aoralscan (AOS). (**C**) TRIOS5 (TRIOS). Statistical significance is indicated by asterisks. (*p < 0.05, ***p < 0.001)

For PS, no significant difference in surface trueness was observed between the full-arch (PS full) and segment-scan (PS segment) conditions (*p* > 0.05) (Fig. 3A). In contrast, for AOS, the segment-scan condition (AOS segment) showed a significantly greater percentage of surface area within ± 50 μm tolerance than the full-arch condition (AOS full; *p* < 0.05) (Fig. 3B). Similarly, for TRIOS, the segment-scan condition (TRIOS segment) demonstrated a significantly higher area within the ± 50 μm tolerance than the full-arch condition (TRIOS full; *p* < 0.001) (Fig. 3C).

### Overall distance deviation

**Fig. 4:**
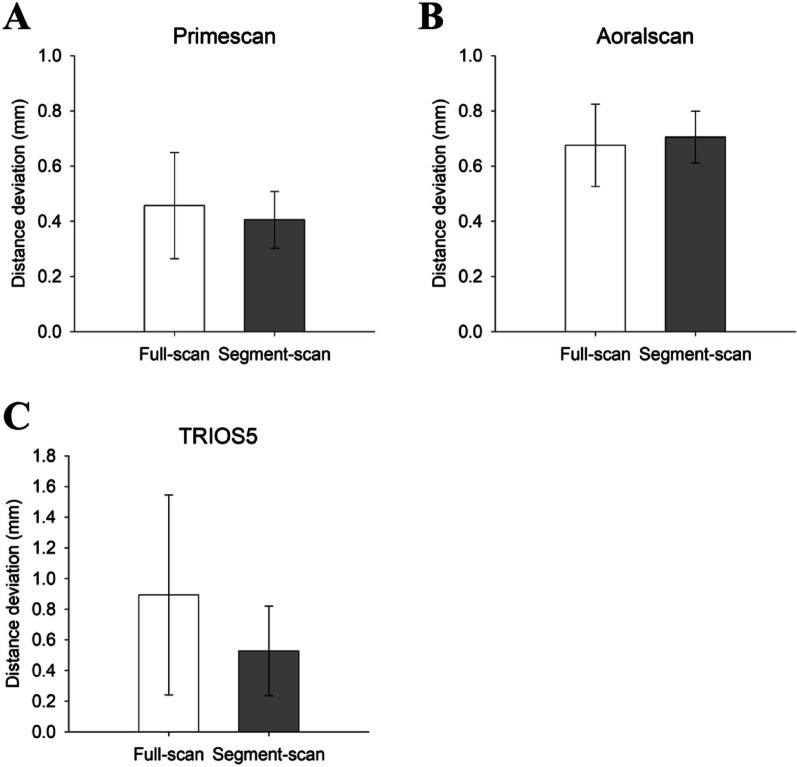
Trueness based on overall distance deviation. Overall distance deviation (Δ*d*_overall_) (*n* = 10 per group). Statistical comparisons between the full-arch and segment scan conditions were performed using the Student’s t-test. (**A**) Primescan (PS). (**B**) Aoralscan (AOS). (**C**) TRIOS5 (TRIOS)

**Fig. 5:**
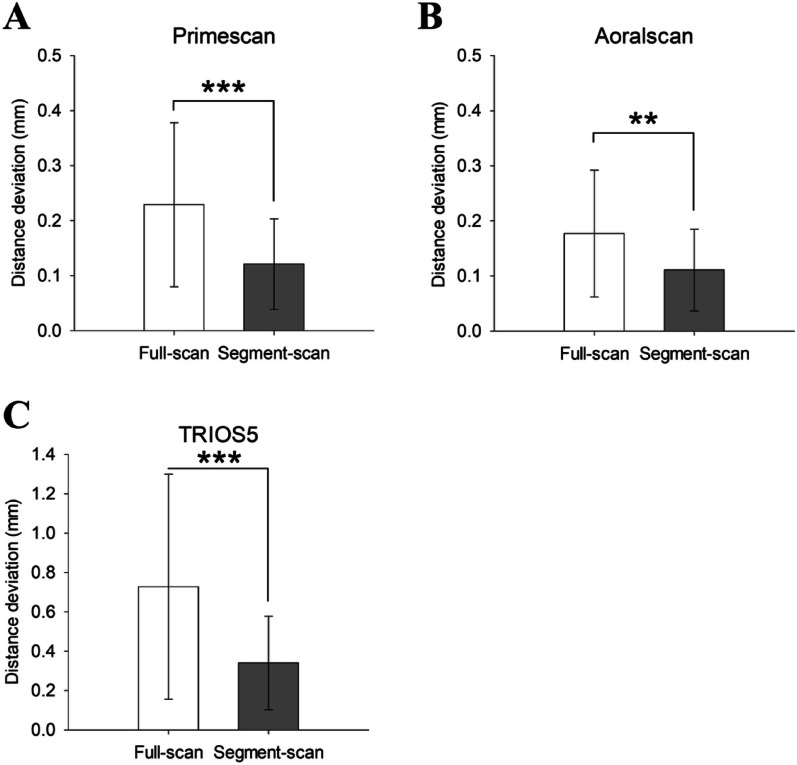
Precision based on overall distance deviation for each intraoral scanner. Precision was calculated as the intra-group pairwise deviation $\:\mid\:{\Delta\:}{d}_{overall,i}-{\Delta\:}{d}_{overall,j}\mid\:\:$and is presented as mean ± standard deviation (n = *C*^2^_10_ = 45 per group). Statistical comparisons between the full-arch and segment-scan conditions were performed using the Mann–Whitney U test. (**A**) Primescan (PS). (**B**) Aoralscan (AOS). (**C**) TRIOS5 (TRIOS). (**p < 0.005, ***p < 0.001)

For overall distance deviation, no significant differences in trueness were observed between the full-scan and segment-scan conditions for PS, AOS, or TRIOS (all *p* > 0.05) (Figs. 4A–C). However, precision was significantly improved under the segment-scan condition compared to the full-scan condition for all evaluated IOSs (all *p* < 0.01) (Tables 1 and 2; Fig. 5A–C).

**Table 1:** Surface trueness, linear trueness, and precision for each scanner and scan range condition

	Area within ± 50 μm (%)	Deviation (Δd_overall_)	Pairwise deviation (|Δd_overall_,i − Δd_overall_,j|)
PS full	88.39 ± 9.84	0.46 ± 0.19	0.23 ± 0.15
PS segment	92.51 ± 5.14	0.41 ± 0.12	0.12 ± 0.08
AOS full	63.46 ± 7.96	0.67 ± 0.15	0.18 ± 0.12
AOS segment	71.34 ± 8.19	0.71 ± 0.09	0.11 ± 0.07
TRIOS full	39.40 ± 10.45	0.89 ± 0.65	0.73 ± 0.57
TRIOS segment	56.66 ± 5.55	0.53 ± 0.29	0.34 ± 0.24

**Table 2:** Statistical comparison between full- and segment-scan conditions

*P* value	Surface agreement	Trueness (Distance deviation)	Precision (Distance deviation)
PS full - PS segment	0.256	0.468	**< 0.001**
AOS full - AOS segment	**0.042**	0.593	**0.009**
TRIOS full – TRIOS segment	**< 0.001**	0.141	**< 0.001**
	(Student’s T-test)	(Student’s T-test)	(Mann–Whitney U test)

The mean and standard deviation of surface trueness, linear trueness, and precision are summarized in Table 1, and the corresponding p-values for comparisons between the full-arch and segment-scan conditions are presented in Table 2. Boldfaces indicate statistical significance between groups.

## Discussion

This study is the first to investigate whether artificially dividing scan body acquisition into three segments and integrating datasets using CAD-based superimposition can improve the accuracy of digital implant scans in an edentulous model. This method makes it possible to exclude movable soft tissue and align only the scan bodies. The results partially rejected the null hypothesis, as segmented scans—except for surface trueness with PS and linear trueness with each scanner—exhibited better accuracy than full-arch scans. Significant differences in surface trueness were observed between the full-arch and segment-scan conditions for AOS and TRIOS.

The accuracy of scans and transfer of implant positions to the working model are paramount for obtaining a passive fit of the implant prosthesis [21]. Inaccurate implant positions in the working model can prevent fabrication of a well-fitting prosthesis, which may exert undesirable stress on the implants, potentially resulting in biological and biomechanical complications, such as screw loosening, bone loss, and ceramic veneer fracture [22]. Researchers have proposed various thresholds for clinically acceptable misfit to prevent biological and biomechanical complications. However, opinions on this topic remain highly controversial [23]. For instance, Jemt reported that misfits up to 150 μm are acceptable [24], whereas Huang et al. suggested a linear displacement of 59–72 μm between implants as an acceptable error range [25]. Di Fiore et al. considered 30–50 μm as the clinically acceptable limit [23]. A recent systematic review further reported that, although no definitive consensus exists regarding the clinically acceptable threshold of misfit, a wide range of tolerable discrepancies has been described, with vertical misfit up to 1 mm and horizontal misfit up to 345 μm being associated with no adverse outcomes [26]. Therefore, the tolerance for surface trueness as a strict standard was set to 50 μm in this study.

The accuracy of digital scans is defined based on precision and trueness. Trueness is defined as the difference between the actual model and recorded digital image when measured in the same manner, whereas precision is the difference between recorded digital images when measured repeatedly. In this study, linear trueness and precision were measured. Since the scan-body library dataset is subsequently matched to CAD data from the digital scan during final prosthesis fabrication, surface trueness was measured to determine the accuracy of each scan body position.

The largest deviation among the inter-scan body distance errors (ΔdAB–ΔdAF) was observed for ΔdAF, which corresponded to the longest inter-scan body span in the model. This finding is consistent with previous reports indicating that scanning errors increase as the distance between scan bodies increases [27].

Although no definitive consensus exists regarding the clinically acceptable threshold of misfit, when the maximum threshold reported in previous studies is used as a reference for interpretation, horizontal misfits of up to approximately 345 μm have been reported not to be associated with adverse biological outcomes [26]. In the present study, the mean deviation of ΔdAF was 0.116 ± 0.062 mm (PS full), 0.120 ± 0.043 mm (PS segment), 0.162 ± 0.078 mm (AOS full), 0.166 ± 0.061 mm (AOS segment), 0.377 ± 0.352 mm (TRIOS full), and 0.171 ± 0.183 mm (TRIOS segment). Whether the deviations remained within the previously reported range of three-dimensional discrepancies that do not exceed bone tolerance thresholds appeared to depend on the compatibility between scanner characteristics, such as depth of field, and scan body geometry.

Variability in reported misfit thresholds may partly be explained by differences in implant–abutment connection geometry and prosthetic screw design. In systems without a centering mechanism of the prosthetic screw, horizontal discrepancies may be partially accommodated without generating excessive stress at the implant–abutment interface, potentially increasing the clinically acceptable level of misfit. In the abutment system used in the present study, the prosthetic screw does not incorporate a centering mechanism. Therefore, interpretation of clinically acceptable misfit values should be made with consideration of the characteristics of the implant system used.

Unlike desktop scanners, which can capture the entire object simultaneously [28], the limited field of view of IOS requires multiple overlapping images that must be “stitched” together using a software algorithm [23]. The IOS merges thousands of images acquired during the scanning process; therefore, intrinsic errors can be generated, particularly during long-span scans. These errors are amplified in edentulous ridges because of the absence of anatomical landmarks and presence of horizontal and vertical distances between scan bodies [29, 30]. To address this problem, artificial landmarks on edentulous ridges [31, 32], scan bodies with extended structures [25, 33], splinting with dental floss or resin [32, 34], and auxiliary geometric devices [35–37] have been proposed. In this study, six implants were placed on both sides of the lateral incisors, first premolars, and first molars. This model is in accordance with the global consensus on clinical guidelines for the rehabilitation of the edentulous maxilla [38]. In clinical situations, the number and position of implants differ from those in this model. However, it can be speculated that the segmentation strategy can reduce the incidence of stitching errors by reducing the volume of photograms in each file.

In this study, segmented scanning and integration using CAD software improved the accuracy of the edentulous ridge model compared with simultaneous full-arch scanning. Moon et al. reported that scanning accuracy differs between full-arch and quadrant-segmented scanning in dentulous patients, particularly in the posterior regions [39]. Therefore, segmented scanning increases accuracy compared to full-arch scanning by allowing partial data acquisition and composition, reducing errors from less accurate molar regions.

Limones et al. reported that voluntary pauses during scanning by pressing the IOS turn-on button improved accuracy by reducing the number of photograms and enhancing the stitching process, whereas involuntary interruption had no significant impact on the accuracy of implant-supported full-arch scans [40]. Lee et al. reported that scan body height influences the accuracy of digital scans, with short scan bodies providing more accuracy than longer ones. They concluded that reducing the distance between the scan body and implant minimizes measurement errors and distortions [41]. Gómez-Polo et al. evaluated scanning patterns for the accuracy of implant-supported full-arch scans [42]. In their novel “O-Lock scanning pattern, the occluso-lingual surfaces of the scan bodies were first recorded, followed by pausing the scan to lock the registered scan body surfaces using the “lock” tool of the IOS software. Subsequently, the interproximal and vestibular surfaces were captured. This approach, which reduced scanning time and the number of photograms, achieved higher accuracy than the “Occlusal-buccal-palatal pass” conventional scan pattern recommended by the manufacturer. Unlike these previous reports, the proposed approach introduces the concept of segmentation based on the scan bodies as reference points. In this strategy, each segment is independently acquired and subsequently aligned using rigid geometric references (scan bodies), rather than relying on soft tissue continuity or extended scan paths. This distinction is critical as it allows for the separation of error sources into intra-segment acquisition and inter-segment alignment errors. Conventional full-arch scanning continuously accumulates stitching errors across the entire arch, whereas the proposed method constrains error propagation within each segment and limits cross-segment error accumulation through geometric registration. This framework enables the interpretation of how stitching-related error propagation contributes differently to trueness and precision in long-span intraoral scans. In the present study, the observation that segmentation mainly improved precision, while showing limited influence on trueness, suggests that this workflow primarily reduces random stitching accumulation errors rather than scanner-specific systematic deviations. Therefore, this approach should be interpreted not only as a procedural modification but also as a strategy to control error propagation pathways during intraoral scanning.

Based on previous reports and the results of this study, reducing the volume of photograms and the incidence of stitching errors is important for enhancing digital scan accuracy. This study was conducted under in vitro conditions; however, in clinical settings, the difficulty of full scans is likely higher than that of segmented scans, due to the presence of tongue and/or buccal mucosa interference, saliva, and patient movement [43]. Therefore, it could be speculated that the superiority of segmented scans may be even greater than the results of this study suggest.

In this study, the scanning strategy had a pronounced effect on precision, whereas only a limited improvement was observed in trueness. The reduction in precision under the full-arch condition is likely attributable to random errors, particularly stitching accumulation errors inherent to intraoral scanning. In contrast, trueness is more strongly influenced by systematic factors such as device calibration and proprietary software algorithms [43]. The segmented scanning strategy likely reduces stitching accumulation errors by limiting error propagation across long-span scans, thereby improving precision without substantially affecting trueness.

This study had some limitations. In this study, a laboratory scanner data was used as the reference data. Therefore, the results from this study should be interpreted as relative comparisons between full-arch and three-segment scanning. The quality of a digital scan with IOS is influenced by many factors, including, the IOS software version, the experience and scanning strategy adopted by the operator, environmental conditions (brightness, temperature, and humidity), patient characteristics (number, position, and inclination of the implant), and the shapes and surface characteristics of the scan body [44]. Therefore, if future software updates for each scanner dramatically improve the accuracy of full-arch scans, the current differences may become less significant. In this study, a well-experienced operator scanned the master model using the same scan-pass method. In an in vitro study, the scanning environment differed from that of the oral cavity, and a dental stone model that reflected light differently was used [45]. Furthermore, this study only evaluated the scanning and data-accumulation steps of the workflow and did not investigate their effects on the downstream process of fabricating the final prosthesis. Further studies are required to assess the scanning and fit of the final prosthesis in a clinical setting.

## Conclusions

Three-segment scanning with subsequent dataset composition is a promising alternative to simultaneous full-arch scanning for digital implant impressions. The main clinical implication of this strategy may be its ability to provide a more consistent full-arch scan acquisition, particularly in situations where error accumulation during long-span scanning is a concern.

## Data Availability

The datasets used and analyzed during the current study are available from the corresponding author on reasonable request.
